# Complete Genome Sequence of *Alteromonas* sp. Strain I4, Isolated from the Japan Sea

**DOI:** 10.1128/MRA.01277-19

**Published:** 2019-11-21

**Authors:** Kentaro Miyazaki, Kouki Izumi

**Affiliations:** a Bioproduction Research Institute, National Institute of Advanced Industrial Science and Technology (AIST), Tsukuba, Ibaraki, Japan; b Department of Computational Biology and Medical Sciences, Graduate School of Frontier Sciences, The University of Tokyo, Kashiwa, Chiba, Japan; c Faculty of Life Sciences, Toyo University, Itakura, Gunma, Japan; University of Southern California

## Abstract

A novel strain of Alteromonas, I4, was isolated from a shallow beach of the Japan Sea. Here, we report the complete genome sequence of I4; this strain contains a single circular chromosome (5,133,645 bp; G+C content, 48.4%) and a single circular putative plasmid (123,836 bp; G+C content, 45.1%).

## ANNOUNCEMENT

Alteromonas is a genus of *Proteobacteria* prevalent in the marine environment (1). To date, many *Alteromonas* species have been proposed (2, 3), with various whole-genome sequencing projects in progress. We collected a seawater sample from a shallow beach in Fukui Prefecture, Japan (36°222′633″N, 136°135′304″E), on 8 August 2019. The sample was spread over Marine agar (Difco) plates and incubated overnight at 37°C. Several colonies were randomly selected, and colony PCR was conducted to amplify the near-full-length 16S rRNA gene (4). DNA sequencing followed by a BLAST search suggested that 6 out of 10 isolates belonged to the genus *Alteromonas*. One of the strains, designated I4, exhibited only 96.6% identity to the closest 16S rRNA gene homologue of Alteromonas lipolytica strain JW12 (NCBI RefSeq accession number NR_156088) and was subjected to whole-genome analysis.

The strain was grown in Marine broth (Difco) at 37°C for 18 h, and genomic DNA was purified using the NucleoBond high-molecular-weight (HMW) DNA kit (TaKaRa Bio), according to the manufacturer’s instructions. Genome analysis was conducted by combining long-read and short-read sequencing technologies. Software analyses were conducted using default parameter settings.

For long-read sequencing, the genomic DNA was passed through the Circulomics short-read eliminator kit; the resultant genomic DNA (1 μg) was used to construct a library using the Native barcoding expansion 1-12 kit (Oxford Nanopore Technologies [ONT]) and ligation sequencing kit (ONT). Sequencing was performed using a GridION X5 system (ONT) with a FLO-MIN106D (R9.4) flow cell (ONT). The data were base called using Guppy v.3.0.3 to yield 275,381 reads (1,208 Mb) with an average length of 4,387 bases during a 10-h runtime, and these were quality filtered (Q ≥ 10; read length, ≥1,000 bases) using NanoFilt v.2.3.0 (5). The longest read length was 136,299 bases, and the *N*_50_ value was 7,650 bases.

For short-read sequencing, a library was constructed using the Nextera DNA library prep kit (Illumina), with an insert length of approximately 350 bp; the library was then subjected to 156-bp paired-end sequencing on the Illumina MiSeq platform. Adapter sequences and low-quality data (Q ≥ 30; read length, ≥10 bases) were trimmed using fastp v.0.20.0 (6); 2.2 million paired-end reads with an average length of 300 bases were obtained.

The long-read and short-read data were assembled using Unicycler v.0.4.7 (7) and polished with Pilon v.1.23 (8), generating a single circular chromosome (5,133,645 bp, 48.5 mol% G+C content) and a single circular putative plasmid (123,836 bp, 45.2 mol% G+C content). Automated annotation was performed using DFAST v.1.1.0 (9). The chromosome contained 4,457 coding sequences, 57 tRNA genes, and 12 rRNA genes, while the putative plasmid contained 83 coding sequences. The coverages of short reads to the chromosome (127.5×) and putative plasmid (123.9×) sequences suggested equal copy numbers for the chromosome and the putative plasmid.

A JSpecies search (10) revealed that I4 showed the highest (69.2%) average nucleotide identity (ANI) with Alteromonas macleodii ATCC 27126 (NCBI RefSeq accession number NC_018632) among the known complete *Alteromonas* genomes. This low ANI value (95% cutoff for the definition of a species [11]) further supports the novelty of strain I4. Moreover, no significant similarity was detected for the putative plasmid.

### Data availability.

The complete genome sequence of *Alteromonas* sp. strain I4 is available from DDBJ/EMBL/GenBank with the accession numbers AP021859 (chromosome) and AP021860 (plasmid). Raw sequencing data were deposited in the DDBJ Sequence Read Archive under the accession number DRA009049 (BioProject number PRJDB8853; BioSample number SAMD00189858).
